# The prevalence of radiographic trochlear dysplasia in patients with patellar fractures

**DOI:** 10.1186/s12891-024-08204-4

**Published:** 2024-12-30

**Authors:** Vanessa Morello, Matthieu Zingg, Philippe Tscholl

**Affiliations:** Division of Orthopaedic and Trauma Surgery, University Hospitals of Geneva, 4 Rue Gabrielle-Perret-Gentil, Geneva, CH-1205 Switzerland

**Keywords:** Trochlear dysplasia, Patellar fracture, Knee, Patello-femoral joint, Patello-femoral dislocation

## Abstract

**Purpose:**

Trochlear dysplasia is found in 3.2% (95% confidence interval (CI) 1.2–6.7) of the general population and linked to patellar instability and anterior cruciate ligament (ACL) injuries. The purpose of this study was to evaluate the prevalence of radiographic trochlear dysplasia in patients with patellar fractures. Secondary outcome was to evaluate the prevalence of trochlear dysplasia in different type of patellar fracture.

**Methods:**

All consecutive 18-year-old or older patients treated in a level-1 trauma center for patellar fracture between 2003 and 2016 were retrospectively evaluated. Trochlear dysplasia was assessed according to Dejour’s classification on a strict lateral knee x-ray. Patellar fractures were analyzed on AP, lateral and axial knee x-rays and classified on pattern and displacement. Patellar dislocation at time of injury was recorded.

**Results:**

Out of 482 patellar fractures, a strict lateral knee x-ray was found in 166 cases (50.6% women, mean age 56years +/-21, 51% right knee). Nineteen cases had trochlear dysplasia (11.5%, 95% CI 6.9–17.9): 10 high-grade trochlear dysplasia meaning type B, C and D (6.0%, 95% CI 2.4–9.7%); 15 without dislocation (9.3%, 95% CI 5.2–15.3). Patellar fracture distribution showed 17 transverse non-displaced (10.2%), 38 transverse displaced (22.9%), 15 avulsion (9%), 27 multifragmented non-displaced (16.3%), 56 multi-fragmented displaced (33.7%), 7 vertical (4.2%) and 6 osteochondral (3.6%) fractures. Four dislocations were recorded, all had osteochondral fractures and trochlear dysplasia. When considering patellar fractures with dislocation, the incidence of osteochondral lesions was significantly higher in patients with trochlear dysplasia (*p* < 0.001). This is not true for any other type of patellar fracture (*p* = 0.138).

**Conclusions:**

This study suggests that there is a significant prevalence of trochlear dysplasia in patients with patellar fractures. This study also suggests that the pattern of patellar fracture is not influenced by the presence of trochlear dysplasia, except for osteochondral fractures associated with patello-femoral dislocations.

## Introduction

 The patello-femoral joint is a sellar joint between the patella and the femoral trochlea and it is stabilized by a combination of bony, ligamentous and muscular restraints. The peak loading stress on the patella occurs at 45° of knee flexion [7, 8, 11, 23].

Trochlear dysplasia is an abnormal morphology of the distal femur characterized by a shallow, flat or even convex trochlea and it is found in about 3.2% (95% confidence interval (CI) 1.2–6.7) of the general population [4, 5].

According to Dejour, trochlear dysplasia is classified in four distinctive types based on x-rays, specifically on a true lateral view of the knee. Type A is characterized by the crossing sign, type B by the supratrochlear spur, type C by the double contour and type D by the ensemble of type A, B and C [6].

Trochlear dysplasia is considered to be a contributing factor to patellar instability and is present in 96% of patello-femoral dislocations [5]. It is also associated to anterior cruciate ligament (ACL) rupture being present in 14.7% (95% CI 10.9–19.2) of ACL injuries [17]. This unexpected association opens to the possibility for other knee trauma pathologies to be associated with trochlear dysplasia.

Patellar fractures account for about 1% of all fractures and its classification is typically descriptive based on fracture pattern and fragments displacement.

Fracture pattern is determined by the injury mechanism (direct or indirect) and it is influenced by many factors including age, gender, bone quality and degree of knee flexion at the time of injury [2, 15, 22].

Direct injury mechanism usually occurs when a blow to the patella generates compressive forces between its anterior and posterior aspect which then comes in contact with the trochlea. In an indirect injury, the fracture is caused by the quadriceps contraction which generates a longitudinal force on the extensor mechanism which translates into (1) a compressive force between the posterior patellar surface and the trochlea and (2) a lateral force due to the Q angle.

The primary objective was to evaluate the prevalence of radiographic trochlear dysplasia in patients with patellar fractures. Secondary objective was to assess each type of patellar fracture prevalence and relate it to the presence of trochlear dysplasia.

To the authors’ knowledge no other study has previously explored the prevalence of radiographic trochlear dysplasia in patients with patellar fractures.

## Materials and methods

### Ethics approval and consent to participate

This study was carried out in accordance with relevant guidelines and regulations and approved by the local ethical commission (Research Ethics Commission of Geneva (CCER « Commission cantonale d’éthique de la recherche »). Informed consent was waived by the local ethical commission (Research Ethics Commission of Geneva (CCER « Commission cantonale d’éthique de la recherche ») due to retrospective nature of the study. Discharge codes were used for patient identification.

### Population

A series of consecutive patients, aged 18 years and older, treated for patellar fracture in a single level-1 trauma center (~ 500’000 inhabitants) between January 2003 and January 2016 were considered for inclusion and retrospectively evaluated. Complete radiographic studies including antero-posterior, true lateral and axial knee x-rays had to be available. Exclusion criteria were underage patients, incomplete (lacking one or more of the three knee x-rays views) or unsatisfying radiographic studies (e.g. lateral x-ray without perfect superimposition of the femoral condyles) or history of previous knee lesion which may alter the trochlear morphology (e.g. distal femur fracture, advanced osteoarthritis, previous patellar trauma or surgery).

### Radiological measurements

**Fig. 1:**
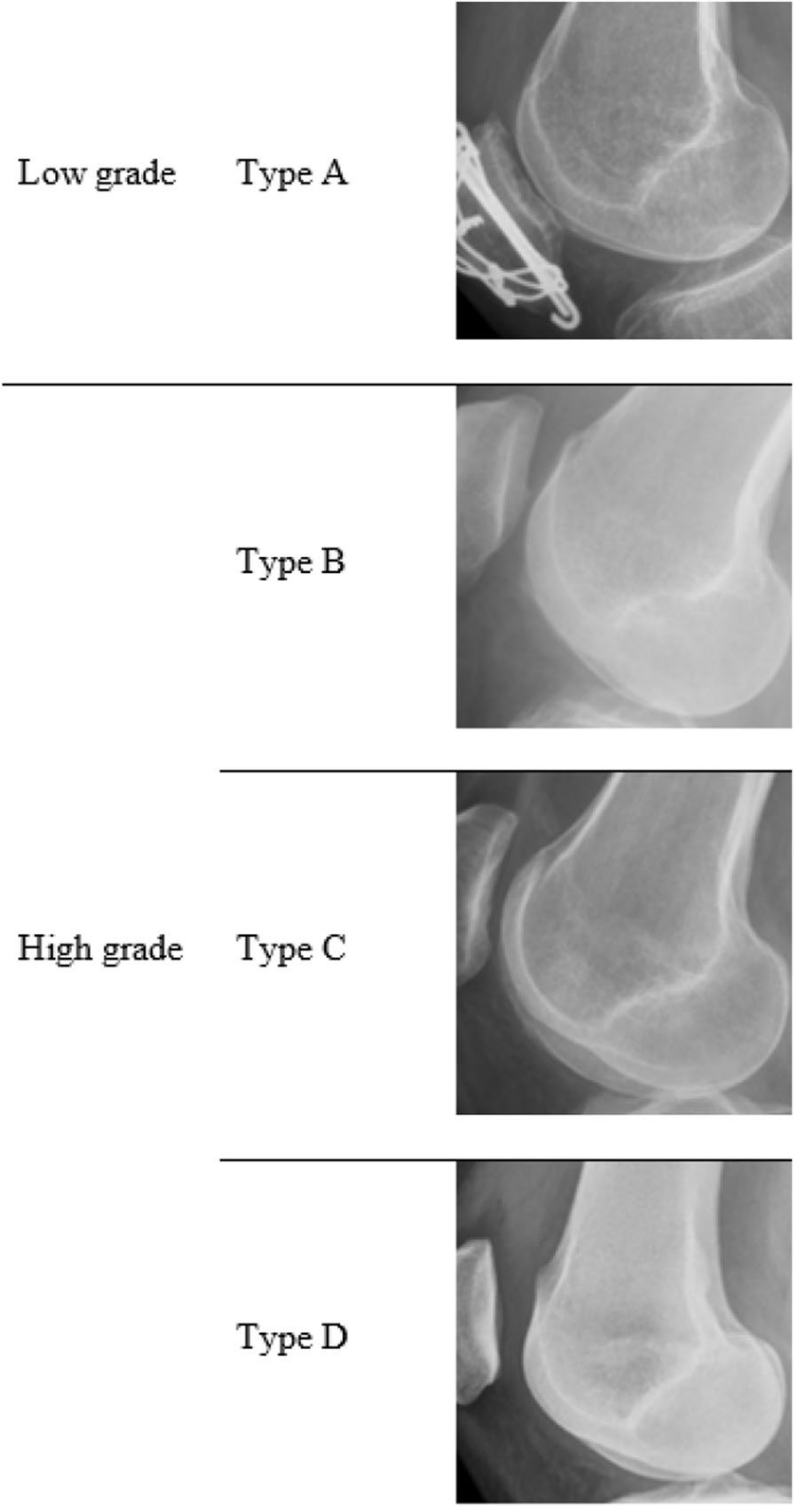
Dejour’s trochlear dysplasia classification

Trochlear dysplasia was assessed according to Dejour’s classification on a true lateral x-ray of the knee in four distinct types, A to D. Dejour’s classification is a reliable and valid tool to analyze radiographic measurements on patellar instability [20]. Images were evaluated by two orthopaedic surgeons specialized in ortho-trauma (VM) and knee-sports (PT). Strict lateral x-rays were considered by the two orthopaedic surgeons when the two femoral condyles were perfectly superimposed. The presence of trochlear dysplasia was classified as low grade (type A) and high grade (type B, C and D) [6]. Both orthopaedic surgeons separately analyzed all images and classified each trochlear dysplasia; when a mismatch in classification was found the case was conjointly reviewed and an agreement was reached. Dejour’s trochlear dysplasia classification on a strict lateral knee x-ray is given in Figure 1. No axial view of the knee was considered for classification because of the lack of reproducibility of it in a traumatic setting.

**Fig. 2:**
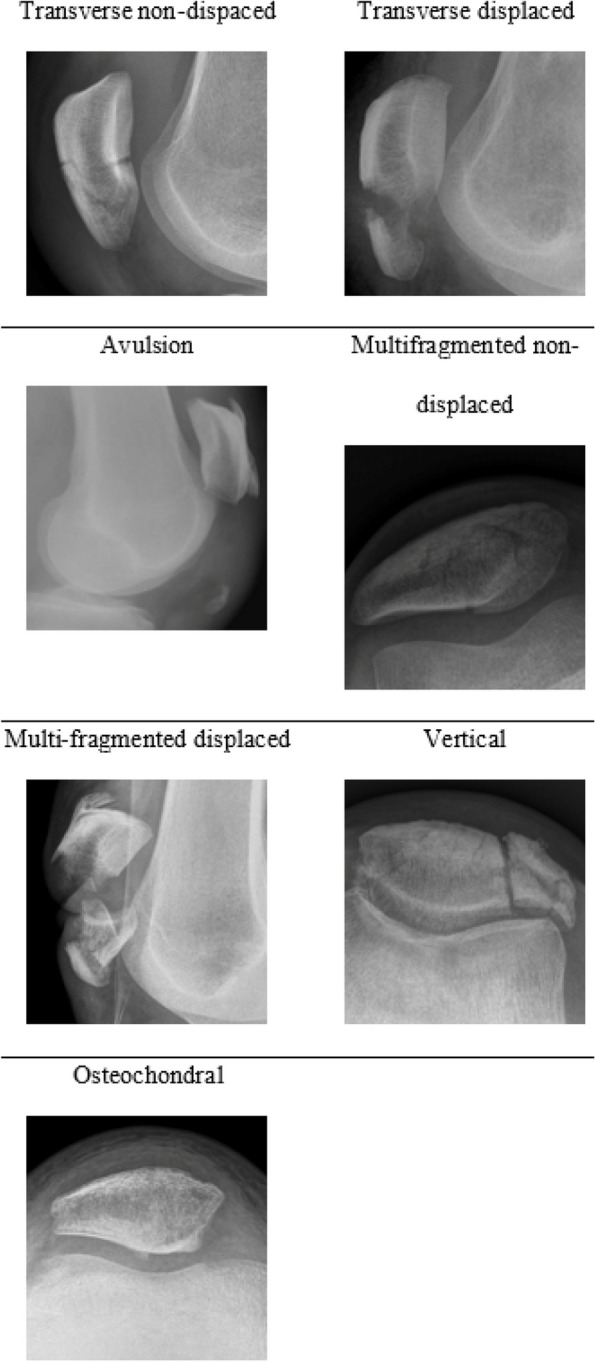
Patellar fracture descriptive classification

Patellar fractures were analyzed on AP, true lateral and axial x-rays of the knee. They were classified on pattern and displacement as transverse non-displaced, transverse displaced, avulsion, multi-fragmented non-displaced, multifragmented displaced, vertical and osteochondral [10]. Both orthopaedic surgeons separately analyzed all images and classified each fracture; when a mismatch in classification was found the case was conjointly reviewed and an agreement was reached. The patellar fracture descriptive classification is given in Figure 2.

Patellar dislocation at the time of injury was also recorded. The prevalence of trochlear dysplasia in patients with patellar fractures was evaluated. Trochlear dysplasia prevalence was compared to patellar fracture type distribution. The same analysis was done with and without patello-femoral dislocations because the presence of trochlear dysplasia associated to patello-femoral dislocations and osteochondral fractures had already been showed in the literature [9, 16, 19, 21].

### Statistical analysis

All statistical analysis were performed using a standard software package (Stata, version 13.1; StataCorp). Continuous variables were summarized as means with standard deviations (SD) and categorical variables were summarized as proportions. Statistical significance was assumed when *p* < 0.05. All confidence intervals (CIs) are 95% CI.

## Results

Four hundred and eighty-two cases of patellar fracture were identified based on discharge diagnostic codes (patellar fracture, patellar dislocation with fracture, patellar fracture-dislocation) and evaluated. Among those, 166 (34.4%) cases met the inclusion criteria and were included in the analysis.

**Fig. 3:**
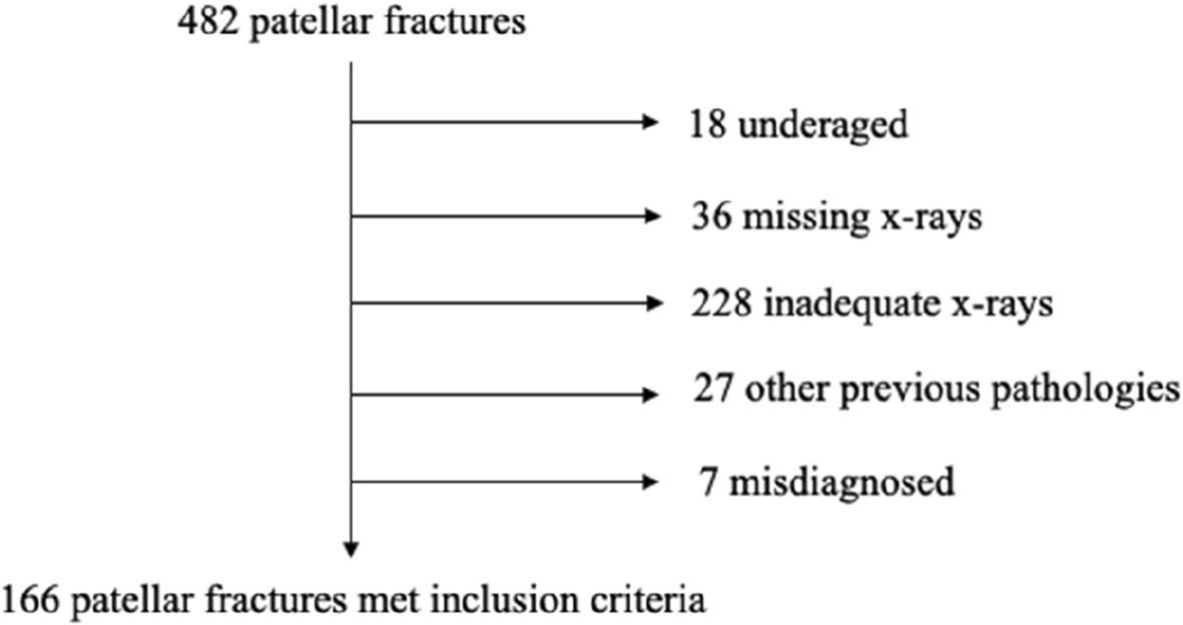
Patient’s selection flow chart

Patient’s selection flow chart is shown in Figure 3.

Demographics are resumed in Table 1.

**Table 1:** Demographics

Gender	84 women (50.6%) ; 82 men (49.4%)
Mean age at injury	56 years old (+- 21 ; min 18 – max 93)
Side	85 right (51.2%) ; 81 left (48.8%)
Number of patellar dislocations	4 (2.4%)

### Trochlear dysplasia prevalence and analysis

Trochlear dysplasia (low- and high-grade) was found in 19 cases with patellar fracture (11.5%, 95% CI 6.9-17.9). High-grade trochlear dysplasia was found in 10 cases (6.0%, 95% CI 2.4-9.7; 3 with type B, 6 with type C and 1 with type D). When considering the patellar fracture population without patello-femoral dislocation, trochlear dysplasia prevalence was 9.3% (15 out of 162 cases, 95% CI 5.2-15.3).

### Patellar fracture type distribution and analysis

Patellar fracture type distribution and trochlear dysplasia presence with and without dislocation are resumed in Table 2. All 4 patients presenting patello-femoral dislocation had trochlear dysplasia and osteochondral fractures. When considering the patellar fracture population with patello-femoral dislocation, the incidence of osteochondral fractures was significantly higher in patients with trochlear dysplasia (*p*<0.001). This was not true for any other type of patellar fracture (*p*=0.138).

**Table 2:** Patellar fracture distribution and trochlear dysplasia presence with and without dislocation

Fracture type	Number (%)	Number with trochlear dysplasia and dislocation (%)		Number with trochlear dysplasia, no dislocation (%)	
		Low and high grade trochlear dysplasia	High grade trochlear dysplasia	Low and high grade trochlear dysplasia	High grade trochlear dysplasia
Transverse non-displaced	17 (10.2)	1 (5.9)	1 (5.9)	1 (5.9)	1 (5.9)
Transverse displaced	38 (22.9)	1 (2.6)	0 (0)	1 (2.6)	0 (0)
Avulsion	15 (9)	2 (13.3)	0 (0)	2 (13.3)	0 (0)
Multifragmented non-displaced	27 (16.3)	3 (11.1)	1 (3.7)	3 (11.1)	1 (3.7)
Multi-fragmented displaced	56 (33.7)	5 (8.9)	3 (5.4)	5 (8.9)	3 (5.4)
Vertical	7 (4.2)	2 (28.6)	1 (14.3)	2 (28.6)	1 (14.3)
Osteochondral	6 (3.6)	5 (83.3)	4 (66.7)	1 (50)	1 (50)
Total	166 (100)	19 (11.5)	10 (6)	15 (9.3)	7 (4.3)

## Discussion

The major finding of this study was the high prevalence of trochlear dysplasia in patients with patellar fracture. The prevalence of trochlear dysplasia related to other knee pathologies has already been documented. Dejour et al. showed that trochlear dysplasia, defined by a positive « crossing sign », was present in 96% of patients with patellar instability. They also showed that trochlear dysplasia was present in only 3.2% (95% CI 1.2-6.7) of the general population based on knee x-rays of an asymptomatic control group comprising 190 patients [5]. Ntagiopoulos et al. showed that the prevalence of trochlear dysplasia in patients with ACL rupture was 14.7% (44 patients out of 299, 95% CI 10.9-19.2), with the same trochlear dysplasia prevalence as Dejour in the healthy control group [17]. The results of this study suggest that the radiological prevalence of trochlear dysplasia in patients with patellar fracture (11.5%) may be higher than in the general population when considering the prevalence found in the literature, and similar to the increased prevalence found in others studies involving knee pathologies [5, 6, 17]. Furthermore, trochlear dysplasia prevalence remains high (9.3%) even when excluding cases of patello-femoral dislocation. This finding may suggest the possibility of an increased stress on the patella when sustaining a possible patellar fracture injury mechanism due to the presence of trochlear dysplasia.

Type of trauma at the time of injury, direct impact on the patella, violent quadriceps contraction or patello-femoral dislocation, implicates a trauma between the posterior surface of the patella and the trochlea. An abnormal morphology of these surfaces may create a different force distribution generating different fracture patterns. Osteochondral fracture have been correlated with patellofemoral dislocations, therefore with trochlear dysplasia, and are known to occur less frequently with direct trauma [9, 16, 19, 21]. This study confirmed osteochondral fracture to be a pattern directly connected to patello-femoral dislocation and trochlear dysplasia (*p* < 0.001). When excluding patello-femoral dislocations from the patellar fracture population, no fracture pattern was found to be statistically significant to be related to trochlear dysplasia (*p* = 0.138). This finding may be correlated to the relatively small number of patients in each subgroup of analysis. Further studies are needed in order to investigate this matter.

The evaluation of the patello-femoral joint is not routinely included in patellar fracture imaging. The need for strict lateral x-ray of the knee strongly limited the number of cases considered (166) when compared to the number of patellar fractures (482). According to the literature the diagnosis of trochlear can be made using x-rays, CT and MR imaging. The standard evaluation of patellar fractures is done with x-rays, and Dejour's classification of trochlear dysplasia, which is based on knee x-rays, has a good reliability [20]. The diagnosis on x-rays requires specifically a true lateral view of the knee with perfectly superimposed posterior femoral condyles: even small femoral rotation (up to 5°) can result in underestimation of the dysplasia. Furthermore the axial view of the knee should not be used because dysplasia occurs in the most proximal part of the femoral groove and the axial view with the knee slightly flexed shows the tangent view of the more distal part of the trochlea giving false-negative results of trochlear dysplasia [1, 3, 7, 12–14, 18, 23]

Patellar dysplasia could not be taken into consideration due to its difficult appreciation after fracture. Its potential presence could have great impact on all analysis and results. Unfortunately, patellar dysplasia needs to be explored with dedicated imaging before any trauma and this was not possible in this study.

Limitations to this study include the lack of a reference group and the decreased number of patients due to the need for appropriate x-rays. The lack of a reference group precluded further statistical analysis which may have increased the significance of these results. The results were although discussed in relation to the data present in the literature. The small number of patients underpowered the results of this study, but it was mainly due to the inappropriate images when considering standards for a true lateral knee x-ray; considering inappropriate x-rays would have compromised the results even more. This limiting factor may also be considered as a bias in selecting a population which did not perfectly reflect the entire population. This fact may also have been increased by the lack of other demographic characteristics.

The main finding of this study was the documentation of yet another significant prevalence of trochlear dysplasia in pathologies around the knee like patello-femoral dislocation, ACL rupture and now patellar fracture. This finding will not alter the course of patellar fracture treatment, but it provides an epidemiologic insight of trochlear dysplasia in patients presenting with patellar fractures.

## Conclusions

This study’s data suggests that there is a significant prevalence of trochlear dysplasia in patients with patellar fractures. This study’s data also suggests that the pattern of patellar fracture is not influenced by the presence of trochlear dysplasia, except for osteochondral fractures associated with patello-femoral dislocation.

## Data Availability

The datasets generated and/or analyzed during the current study are not publicly available because due to the restrictions applied, but are available from the corresponding author on reasonable request and CCER approval (data are de-identified).

## Abbreviations

ACL: Anterior cruciate ligament
CI: Confidence interval
SD: Standard deviation
